# Author Correction: Archaean continental crust formed from mafic cumulates

**DOI:** 10.1038/s41467-024-45941-5

**Published:** 2024-02-13

**Authors:** Matthijs A. Smit, Kira A. Musiyachenko, Jeroen Goumans

**Affiliations:** 1 Department of Earth, Ocean and Atmospheric Sciences, University of British Columbia, 2020-2207 Main Mall, Vancouver, V6T 1Z4 Canada; 2 Department of Geosciences, Swedish Museum of Natural History, Frescativägen 40, SE-104 05, Stockholm, Sweden

**Keywords:** Geochemistry, Precambrian geology, Petrology, Geodynamics, Tectonics

Correction to: *Nature Communications* 10.1038/s41467-024-44849-4, published online 24 January 2024

The original version of this article contained an error in reference numbering in the Introduction and the Results and Discussion section, which incorrectly read ‘*An example of obscured cumulate sources is provided by TTGs from the Pilbara Craton*^*22*^’; and ‘*Hydrous basaltic rocks are generally considered*^*8,11,12,22,24*^’. The correct version states ‘23’ in place of ‘22’. This has been corrected in both the PDF and HTML versions of the Article.

