# Supplementary material for: Archaean continental crust formed from mafic cumulates
Source: Nat Commun. 2024 Jan 24;15:692. doi: 10.1038/s41467-024-44849-4 (PMC10808207; doi:10.1038/s41467-024-44849-4)
Supplement: Supplementary file 1 — Supplementary Information [file 41467_2024_44849_MOESM1_ESM.pdf]

Supplementary Note 1 – The HFSE systematics of granitoids

To build a geochemical reference frame for TTGs we explored the Ti*-Nb systematics of granitoid rocks from various settings (Fig. S1). Granitoids from collisional orogens, and active margins define a Nb-Ti_N/Ti* data continuum anchored in a composition that – with Ti_N/Ti* of c. 1 and a Nb concentration of 8 ppm – resembles the average composition of the bulk continental crust, as well as upper continental crust[1]. On the low-Nb side, granitoid compositions trend away from this average granitoid end-member (GEM) composition. The data follow a rutile-dominated fractionation trend of decreasing Nb with decreasing Ti_N/Ti*. This trend shows that these granitoids were ultimately derived from rutile-bearing sources with GEM composition, either by single-stage partial melting of GEM at different degrees or by multiple stages of crystallisation and re-melting of GEM-derived melts. Adakites follow this same trend, reflecting their petrogenesis as partial melts from subducted rutile-stable sediments and mafic crust[2].

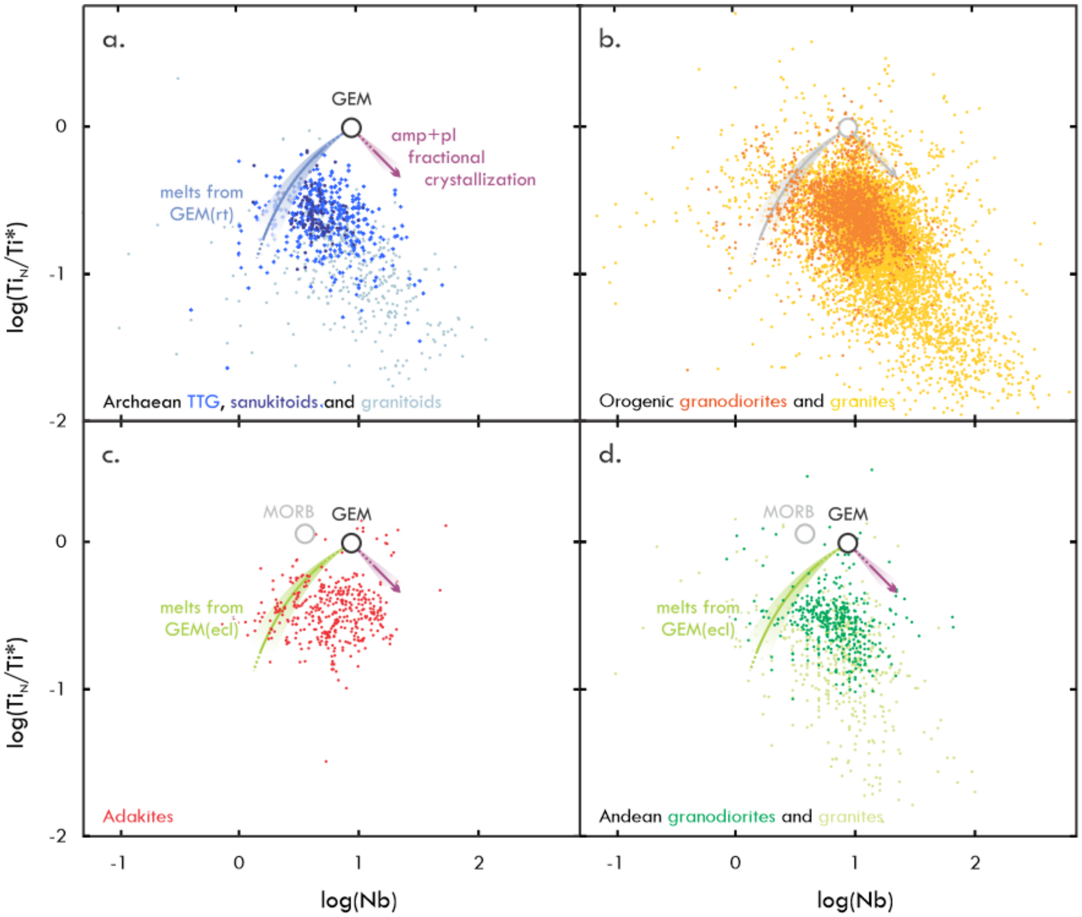

Fig. S1: Ti_N/Ti* versus Nb concentration for granitoids shown separately: a) Archaean TTGs[3], sanukitoids[4] and granitoids, b) granitoids from collisional orogens and other orogenic settings, c) adakites, and d) granitoids from Andean margins. The following chemical vectors are shown for reference where relevant: melt compositions for rutile-bearing sources, eclogite and granulite combined (a, b), and for eclogitic sources (c, d), and crystal fractionation curves assuming a generic assemblage of amphibole and plagioclase (1:1). MORB[5] is shown for reference for adakites and Andean granitoids. Absence of samples with (Ti_N/Ti*)/Nb higher than those of orogenic granitoids indicates that any slab melting components in these rocks likely involves pelagic sediments (similar to GEM) rather than MORB.

With decreasing Ti_N/Ti^*, the granitoids that approximate the residual rutile trend show gradually increasing SiO_2, and decreasing FeO, MgO, TiO_2, CaO and P_2O_5 (Fig. S2). The compositional variation among these rocks thus appears to largely reflect differences in the degree of melting, rather than differences in source composition, with the highest Ti_N/Ti^* representing the highest-possible degree of melting involved in granitoid magma tism. These compositional changes apply to all granitoids, regardless of their tectonic setting and likely reflect progressively lower degrees of melting. Evolved granitoids are enriched in Nb relative to granitoids that plot on the residual-rutile trend and define a compositional continuum that captures many processes, from crystal fractionation of amphibole and plagioclase, to (re-)melting at various depths, possibly involving sources that contain titanite or ilmenite, instead of rutile, or are entirely free of titaniferous phases. This continuum provides a predictable framework for interpreting granitoid compositions in terms of primary source composition and the degree of chemical change superimposed by re-melting and crystal fractionation.

Supplementary Note 2 – Source REE signatures

Unfractionated low-*F* TTGs exhibit high La/Sm, Sm/Yb and Sr/Y, especially those with low Ti_N/Ti^* and MgO. Modal modelling was done to investigate whether these values are solely due to element partitioning during partial melting of a source with MORB-like REE systematics, or whether the source has fractionated REE. The calculations (see Methods) show that a garnet-bearing amphibolite source ($grt_{25}amp_{40}pl_{30}rt_{10}$) would produce melts with 2.7-times higher La/Sm and 3.1-times higher Sm/Yb than the source at the minimum degree of TTG melting (10%[6]). These factors are 2.2 and 4.1 for a realistic mafic-granulite assemblage ($grt_{25}cpx_{40}pl_{30}rt_5$), and 3.3 and 3.8 for hydrous eclogite ($grt_{40}cpx_{45}amp_{10}rt_5$). Even though these factors are considerable, they do not alone explain the fractionated REE composition of some of the low-*F* TTGs (Fig. 3). These compositions are so fractionated that they require garnet-stable melting of a source with La/Sm and Sm/Yb of at least 7.8 and 2.5, respectively. As an extreme case, we consider a scenario of non-modal melting (see Methods) of a MORB source comprising a realistic mafic-granulite assemblage and plagioclase contributing more than 50% to the melt. This scenario may not be realistic, as it would provide significantly lower Rb/La and La/Yb, and higher REE concentrations in the melt than are observed. Regardless, even in that case, La/Sm and Sm/Yb are not as high as observed in some low-*F* TTGs. The source of TTGs thus must have had fractionated REE signatures with La/Sm and Sm/Yb higher than that of MORB.

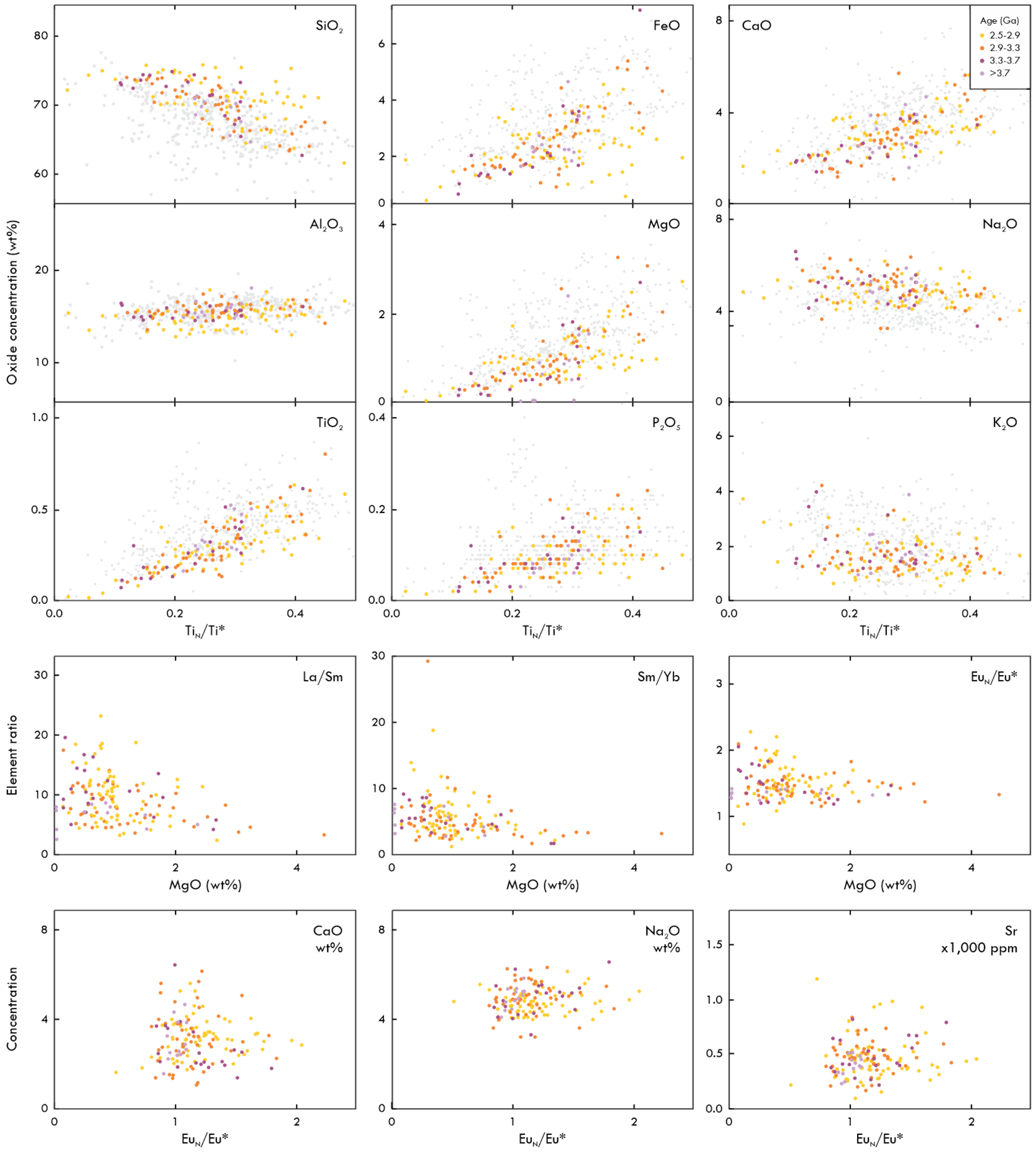

Fig. S2: Major-element concentrations, La/Sm, Sm/Yb and Eu_N/Eu^* versus Ti_N/Ti^*, and CaO, Na_2O and Sr concentrations versus Eu_N/Eu^* for relatively unfractionated TTGs ($F < 5$). Normalisation is to Primitive Mantle[5] or, for REE, to the composition of CI chondrite[6]. Data for other granitoids in grey. Melts with large Ti deficiencies are generally rich in SiO_2 and poor in FeO, MgO, TiO_2, CaO and P_2O_5. No systematic difference in CaO, Na_2O and Sr concentration is observed for TTGs.

Supplementary Note 3 – Melts derived from mafic cumulate rocks

To test whether TTGs can be derived from gabbro(-norite), melt modeling was done involving such composition and plausible source mineral assemblages. A representative average composition of global gabbros and gabbronorites was taken as input. Although these rocks in general differ significantly in composition, they are – with the exception of Rb-Th, Sr and Pb – comparable in their incompatible-element concentrations. The central 40% of composition observed for gabbros (*n* = 8,153; https://georoc.eu/) and gabbronorites (*n* = 697) were used in a batch-melting model (Methods, all partition coefficients in Table S3). Garnet-bearing amphibolite (grt$_{25}$amp$_{40}$pl$_{30}$rt$_{5}$), mafic granulite (grt$_{25}$cpx$_{40}$pl$_{30}$rt$_{5}$), and eclogite assemblages (grt$_{40}$cpx$_{45}$amp$_{10}$rt$_{5}$) were tested. We note that each of these is possible, because gabbroic rocks within the lower crust may contain substantial amounts of H_2O. For the 1,852 samples for which H_2O concentrations are available, the central 95% of H_2O concentrations is between 0.02-4.50 wt%, with an average of 2.3 wt%, with no clear differences between gabbros from convergent margins or from orogenic settings. These concentrations likely reflect primary hydrous minerals – presumably amphibole and phlogopite – as there is no correlation between H_2O concentration and the chemical index of alteration.

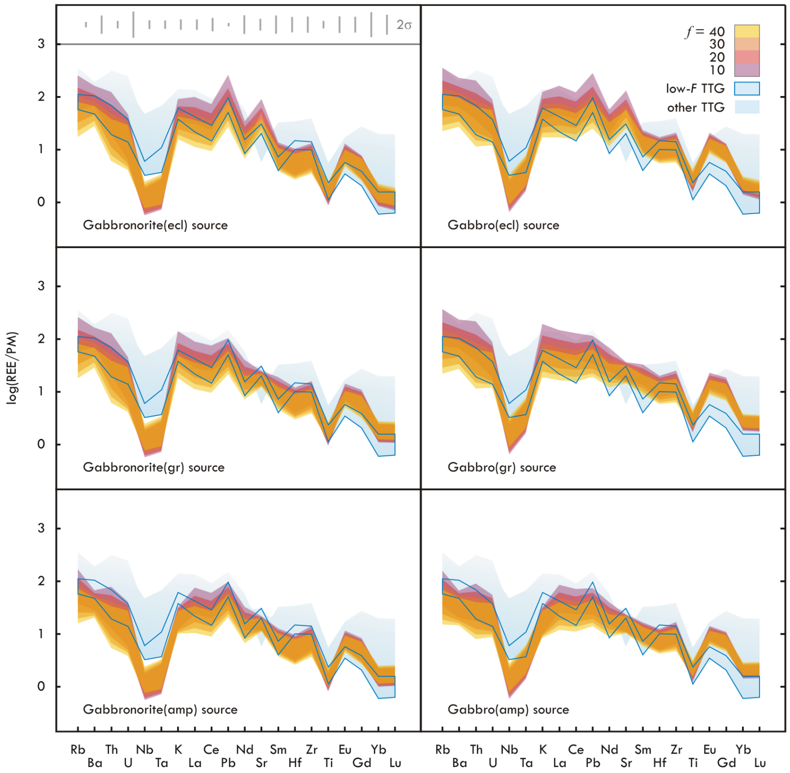

Fig. S3: Model TTG melts produced at different degrees of melting (*f*) from gabbronorite and gabbro sources with eclogitic (ecl; grt$_{40}$cpx$_{45}$amp$_{10}$rt$_{5}$), granulitic (gr; grt$_{25}$cpx$_{40}$pl$_{30}$rt$_{5}$) and amphibolite (amp; grt$_{25}$amp$_{40}$pl$_{30}$rt$_{5}$) mineral assemblages. Gabbronorite and gabbro compositional ranges used in the calculations cover the central 50% of compositions observed for these rocks (https://georoc.eu/). All source lithologies broadly provide a match between model melt product and low-*F* (<5) TTG compositions, with eclogitic sources also having the best fit through its distinct positive Pb and Sr anomalies. The 2σ error bars in the top left diagram provide a representative estimate of uncertainties stemming from partition coefficients.

The modelled melts produced by 10-40% melting of these compositions and mineral assemblages have incompatible-element compositions that strongly resemble those observed in primitive TTGs (Fig. S3). Systematic differences only occur for Nb, Ta, Zr, and Hf. Where significant, these differences may indicate non-modal melting of the refractory accessory minerals that sequester these elements (rutile, zircon) or underestimated uncertainties in partition coefficients, rather than a significant discrepancy between model and observation. A gabbro(-norite) with a granulitic mineral assemblage of garnet, clinopyroxene and plagioclase provides a better fit between model melt and TTG parental melt than such a source rock comprising garnet, amphibole and plagioclase. Better than either is the match obtained between low-*F* TTGs and model melt from a hydrous eclogite source assemblage comprising garnet, clinopyroxene and trace amphibole (Fig. S3). The absence of plagioclase in this assemblage makes Sr and Pb significantly more incompatible, thus amplifying Sr and Pb excess of gabbroic sources to those observed in primitive TTGs. Melt modelling thus confirms the validity of the melting of a meta-gabbro(-norite) source in the petrogenesis of TTGs.

References

[1] Rudnick, R.L. and Gao, S. Composition of the Continental Crust. *In* Treatise on Geochemistry v. 3 (Eds Holland, H.D., Turekian, K.K.). Elsevier, Amsterdam, pp. 1-64 (2003). https://doi.org/10.1016/B0-08-043751-6/03016-4

[2] Castillo, P.R., Adakite petrogenesis. *Lithos* **134-135**, 304-316. https://doi.org/10.1016/j.lithos.2011.09.013

[3] Johnson T.E. Kirkland, C.L., Gardiner, N.J. *et al.* Secular change in TTG compositions: Implications for the evolution of Archaean geodynamic. *Earth Planet. Sci. Lett.* **505**, 65-75 (2019). https://doi.org/10.1016/j.epsl.2018.10.022

[4] Heilimo, E., Halla, J., Hölttä, P., Discrimination and origin of the sanukitoid series: Geochemical constraints from the Neoarchean western Karelian Province (Finland). *Lithos* **115**, 27-39 (2010). https://doi.org/10.1016/j.lithos.2009.11.001

[5] Hofmann, A.W., Chemical differentiation of the Earth: the relationship between mantle, continental crust, and oceanic crust. *Earth Planet. Sci. Lett.* **90**, 297-314 (1988). https://doi.org/10.1016/0012-821X(88)90132-X

[6] Boynton, W.V. Cosmochemistry of the rare earth elements; meteorite studies. *In* Developments in Geochemistry v. 2: Rare earth element geochemistry (Ed. Henderson, P.), Elsevier, Amsterdam. pp. 63-114 https://doi.org/10.1016/B978-0-444-42148-7.50008-3
