# Supplementary material for: Archaean continental crust formed from mafic cumulates
Source: Nat Commun. 2024 Jan 24;15:692. doi: 10.1038/s41467-024-44849-4 (PMC10808207; doi:10.1038/s41467-024-44849-4)
Supplement: Supplementary file 3 — Description of Additional Supplementary Files [file 41467_2024_44849_MOESM3_ESM.pdf]

<u>Description of Additional Supplementary Files</u>

Supplementary Data 1: Partition coefficients (D) for Nb, Zr and Gd in rutile, titanite and ilmenite in basaltic-andesitic systems.

Supplementary Data 2: Partition coefficients (D) for Ti, Nb, Zr and Gd in amphibole, clinopyroxene, clinopyroxene and plagioclase in basaltic-andesitic systems.

Supplementary Data 3: Partition coefficients (D) for trace elements in amphibole, clinopyroxene, garnet and plagioclase in basaltic systems used to model source compositions.
